# Digital Twin Model for Predicting Hygrothermal Performance of Building Materials from Moisture Permeability Tests

**DOI:** 10.3390/ma18184360

**Published:** 2025-09-18

**Authors:** Anna Szymczak-Graczyk, Jacek Korentz, Tomasz Garbowski

**Affiliations:** 1 Department of Construction and Geoengineering, Poznan University of Life Sciences, Piątkowska 94 E, 60-649 Poznan, Poland; anna.szymczak-graczyk@up.poznan.pl; 2 Institute of Civil Engineering, University of Zielona Gora, Prof. Z. Szafrana 1, 65-516 Zielona Góra, Poland; j.korentz@ib.uz.zgora.pl; 3 Department of Biosystems Engineering, Poznan University of Life Sciences, Wojska Polskiego 50, 60-627 Poznan, Poland

**Keywords:** moisture permeability, digital twin, hygrothermal simulation, building materials durability, capillary, diffusion transport

## Abstract

Moisture transport in building materials significantly influences their durability, mechanical integrity, and thermal performance. This study presents an experimental investigation of moisture permeability in a range of traditional and modern wall elements, including autoclaved aerated concrete (ACC), ceramic blocks, silicate blocks, perlite concrete blocks, and concrete units. Both vapor diffusion and capillary transport mechanisms were analyzed under controlled climatic conditions using gravimetric and hygrometric methods. Among the tested materials, autoclaved aerated concrete (AAC) was selected for detailed numerical modeling because of its high porosity, strong capillarity, and widespread use in modern construction, which make it especially vulnerable to moisture-related degradation. Based on the experimental findings, a digital twin was developed to simulate hygrothermal behavior of walls made of ACC under various environmental conditions. The model incorporates advanced moisture transport equations, capturing diffusion and capillary effects while considering real-world variables, such as relative humidity, temperature fluctuations, and wetting–drying cycles. Calibration demonstrated strong agreement with experimental data, enabling reliable predictions of moisture behavior over extended exposure scenarios. This integrated approach provides a robust engineering tool for assessing the long-term material performance of AAC, predicting degradation risks, and optimizing material selection in humid climates. The study illustrates how coupling experimental data with digital modeling can enhance the design of moisture-resistant and durable building envelopes.

## 1. Introduction

Moisture transport in building materials plays a fundamental role in their durability, mechanical integrity, and the overall energy efficiency of buildings [1,2,3,4]. Moisture penetration into materials can trigger a series of adverse phenomena, including reduced mechanical strength and damage resistance [5], increased thermal conductivity, and consequently, greater heat loss through building envelopes [6,7], which directly translates into higher energy demands and operating costs [8]. Elevated moisture levels also promote the growth of microorganisms, such as mold and fungi, which negatively affect both occupant health and the long-term durability of structural components [9,10]. The problem of moisture accumulation in building envelopes has gained particular significance in light of current design trends focused on maximizing airtightness to reduce energy losses [11,12]. However, these solutions limit the potential for natural air exchange and moisture evacuation, which—when combined with intensive building use, increased indoor humidity generation, accelerated construction schedules, and the elimination of material seasoning—can lead to long-term moisture buildup within partitions [13]. Additionally, ongoing climate changes and the increasing frequency of extreme weather events, such as heavy rainfall and prolonged periods of high relative humidity, pose significant challenges to material durability and structural safety [14,15].

Traditional methods for assessing moisture transport in building materials, such as the Glaser method [16], are based on steady-state assumptions and fail to account for the nonlinear and dynamic processes occurring in porous construction materials, including water vapor diffusion under variable climatic conditions, capillary moisture transport, and phase change effects [17,18,19]. In response to these limitations, advanced numerical modeling techniques have been developed, including coupled heat and moisture transport simulations, such as those conducted using WUFI 2D software [20,21], which incorporate real meteorological data and dynamic interactions within building components. Despite their accuracy, such methods are time-consuming and computationally intensive, which limits their practicality for multivariable analysis, optimization, and the design of moisture-resilient materials [22,23].

In recent years, the concept of the digital twin has gained traction in material and building engineering as a tool for integrating experimental data with numerical simulations [24,25,26,27,28,29]. This approach enables accurate replication of building material behavior under real-world conditions and facilitates the prediction of responses to changing environmental factors [30]. Digital twins incorporate complex processes, such as vapor diffusion, capillary transport, and coupled hygrothermal phenomena, providing engineers and designers with effective tools for optimizing material selection and designing structures resistant to moisture-related degradation [31,32,33].

This paper presents the results of experimental moisture permeability tests conducted on wall elements made of autoclaved aerated concrete, ceramic hollow bricks, sand–lime blocks, perlite concrete, and traditional concrete. These materials were chosen because they represent the most common categories of masonry elements used in Central European construction, ranging from highly porous and lightweight blocks to dense concrete units. Such diversity enabled us to capture a wide spectrum of moisture transport behaviors, from pronounced capillary suction in porous materials (e.g., AAC, perlite concrete) to limited absorption in dense silicate and concrete blocks. Including these materials also allowed us to evaluate and compare their performance under identical boundary conditions, providing a relevant benchmark for both practical applications and model calibration.

Both vapor diffusion and capillary transport mechanisms were analyzed under controlled conditions using gravimetric and hygrometric methods [13,34]. Based on the experimental results, a digital twin model was developed to simulate the hygrothermal behavior of materials under varying environmental conditions. Model calibration showed excellent agreement with experimental data, confirming the effectiveness of the approach for predicting moisture behavior in untested materials and across diverse operating scenarios.

While several wall materials were experimentally examined, a digital twin was specifically developed for autoclaved aerated concrete, with the remaining results serving as contextual benchmarks. Therefore, the aim of this study was to develop and validate a digital twin that enables accurate modeling of moisture transport phenomena in ACC walls based on experimental results and to assess its applicability for predicting hygrothermal behavior under changing climatic and operational conditions. This framework can serve as a foundation for designing durable, energy-efficient, and moisture-resistant building envelopes.

## 2. Materials and Methods

### 2.1. Experimental Setup

Five different building materials were selected for the study, assuming they represent typical elements used for constructing exterior walls of buildings. The materials included concrete blocks, autoclaved aerated concrete blocks, perlite concrete blocks, Sika sand–lime blocks, and ceramic hollow bricks. Each wall specimen was finished on the interior side with a standard gypsum plasterboard (type A according to [Hygrothermal performance of building components and building elements—Assessment of moisture transfer by numerical simulation 520 (EN) 520] of 12.5 mm thickness, representing a typical internal wall lining.

**Figure 1:**
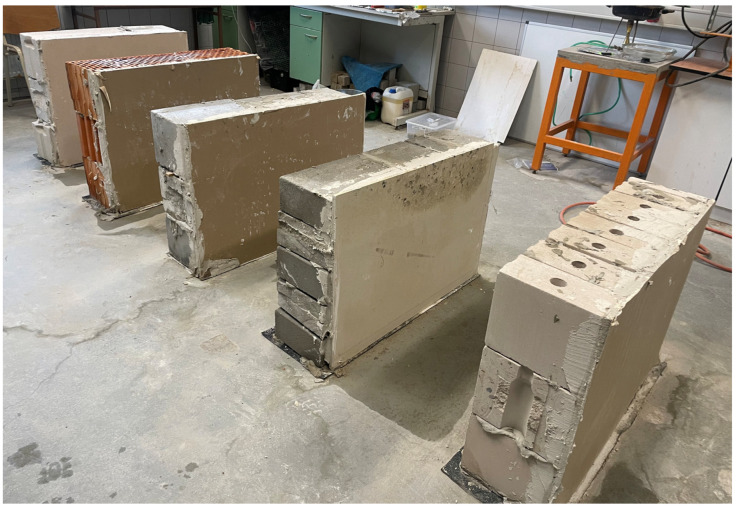
Walls selected for the study. From left to right: wall made of autoclaved aerated concrete, wall made of ceramic hollow blocks, wall made of perlite concrete, wall made of concrete blocks, and wall made of Sika sand–lime blocks.

Figure 1 presents the laboratory test setup. During the tests, the laboratory conditions were maintained at a constant temperature of approximately 14 °C and relative humidity of around 60%. Each wall was sprayed with water twice daily, with each irrigation lasting approximately 8 min. During each irrigation cycle, approximately 0.9–1.0 L of water per square meter of wall surface was applied, which ensured complete wetting of the exterior face without continuous runoff. For each material type, six wall specimens (*n* = 6) were prepared and tested under identical boundary conditions. This sample size ensured reproducibility of the results and provided a reliable basis for numerical model calibration.

### 2.2. Theoretical Background

To simulate the long-term effects of atmospheric precipitation, the exterior surfaces of the walls were subjected to controlled artificial rain exposure. The applied amount of water was regulated to ensure complete wetting of the outer surface without inducing continuous runoff, thereby promoting water absorption rather than drainage.

Moisture transport in porous building materials is a complex process involving the coexistence of several mechanisms: vapor diffusion, capillary transport of liquid water, and moisture movement driven by water vapor pressure gradients. In the present case of unidirectional wetting through surface contact with water, capillary transport is the dominant mechanism. However, vapor diffusion within the pore network is also taken into account.

Water penetrates porous materials due to capillary rise. The mathematical description is based on the Richards equation, which combines the continuity equation with Darcy’s law (1) [35]:

(1) $$\frac{\partial \theta }{\partial t}=\nabla \left[K(\theta )\nabla (h+z)\right],$$

where

*θ*—volumetric moisture content [m^3^/m^3^];
*K*(*θ*)—hydraulic conductivity [m/s], dependent on moisture content;
*h*—capillary pressure head [m];
*t*—time [s];
*z*—vertical coordinate [m].

For simplified cases (e.g., under low moisture conditions), moisture transport can be approximated by the moisture diffusion equation based on Fick’s second law (2) [35]:

(2) $$\frac{\partial c}{\partial t}=D\frac{\partial^{2}c}{{\partial x}^{2}},$$

where

*c*—water vapor concentration or moisture content [kg/m^3^];
*t*—time [s];
*x*—depth within the material [m];
*D*—moisture diffusion coefficient in the material [m^2^/s].

In the case of building materials, the diffusion coefficient *D* may depend on relative humidity, temperature, and the pore structure of the material (including micro-, meso-, and macropores). Fick’s second law explains that moisture content within a material tends toward equilibrium. For example, after rainfall exposure ceases, moisture migrates from wetter layers toward drier ones, while during prolonged wetting, moisture gradually penetrates deeper into the material.

### 2.3. Methods for Measuring Moisture Content in Building Materials

Moisture testing was conducted using both destructive (direct) and non-destructive (indirect) methods. The aim was to determine the moisture distribution in autoclaved aerated concrete walls after prolonged exposure to atmospheric precipitation. The study employed various electrical moisture meters: dielectric, resistance-based, and microwave devices, as well as the gravimetric method. Core samples for the destructive, oven-drying method were collected using a 20 mm diameter drill bit. Samples were taken from two depths in the wall: approximately 5 cm and 10 cm. The specimens were weighed in their wet state, then dried at 105 °C for 24 h, and weighed again. The moisture content was calculated according to Equation (3) [35]:

(3) $$w=\frac{m_{wet}-m_{dry}}{m_{dry}}\cdot 100\%,$$

For non-destructive moisture measurements, three different instruments were used: the Trotec T610 microwave moisture meter (Trotec, Heinsberg, Germany), which enables moisture assessment up to a depth of several centimeters based on the absorption of electromagnetic waves; the Brennenstuhl MD resistance meter (Hugo Brennenstuhl GmbH, Tübingen, Germany), which measures the electrical resistance between electrodes inserted into the material; and the Gann dielectric moisture meter (Gann Mess- u. Regeltechnik GmbH, Gerlingen, Germany), which determines the material’s moisture content by measuring its capacitance. Measurements were taken at heights of 10, 40, and 60 cm, allowing for an evaluation of the vertical moisture distribution and the impact of floor-level water contact. The use of a set of methods based on different measurement principles enabled non-destructive verification of both surface and subsurface moisture levels, optimal placement of the core sampling points, and comparison of indirect measurement data with results obtained using the reference method (oven-drying).

### 2.4. Numerical Model and Implementation

#### 2.4.1. Physical Background and Governing Equation

Moisture transport in porous building materials is strongly influenced by the nonlinear dependence of diffusivity on saturation as well as by anisotropy induced by the material structure. Similar approaches accounting for nonlinear and anisotropic diffusion have been reported in the literature, confirming the need to move beyond simplified linear Fickian formulations [17,19,21,34]. In particular, Tariku et al. [17] and Pel et al. [34] emphasized that classical steady-state models underestimate moisture penetration in porous walls, while Hagentoft [19] demonstrated that anisotropy arising from reinforcement or casting layers can substantially affect long-term predictions. Our formulation builds on these established findings, extending them with explicit calibration against experimental AAC data.

In real-life engineering conditions, concrete walls (e.g., basement walls, retaining structures, or outer building envelopes) are subjected to long-term and spatially variable moisture exposure. Modeling moisture transport in such structures requires both nonlinear and anisotropic formulations due to the physical characteristics of the material and the underlying processes.

Nonlinearity arises from the strong dependence of moisture diffusivity on the saturation level. Dry concrete significantly impedes moisture transport compared to wet concrete. Experimental data and standards (such as [Hygrothermal performance of building components and building elements—Assessment of moisture transfer by numerical simulation 15026 (EN) 15026] or [Wissenschaftlich-Technische Arbeitsgemeinschaft für Bauwerkserhaltung und Denkmalpflege (WTA) guidelines]) often define exponential or power-law relations for D(w), which better reflect the true dynamics of wetting and drying in cementitious materials.

Anisotropy, on the other hand, is a consequence of direction-dependent features in the concrete structure, such as reinforcement, casting layers, preferential cracking, or formwork effects. These factors often result in faster horizontal transport (e.g., along reinforcement layers or technological interfaces) compared to the vertical direction. Ignoring such behavior can lead to misprediction of drying rates, localized moisture accumulation, and subsequent degradation risks.

By applying a model with nonlinear and tensorial anisotropic diffusivity, it is possible to better simulate long-term moisture evolution in reinforced concrete walls and more accurately assess risks related to durability, energy performance, and moisture damage.

Moisture transport in porous construction materials, such as concrete, occurs primarily via vapor diffusion and capillary flow within the pore structure. Under isothermal conditions and assuming the absence of forced convection, the process is governed by the nonlinear moisture diffusion equation [17,19,34]:

(4) $$\frac{\partial w}{\partial t}=\nabla \left(D\left(w\right)\nabla w\right),$$

where

- $w\left(x,y,t\right)$ is the local moisture content [kg/m^3^];
- D(w) is the nonlinear diffusivity tensor [m^2^/s];
- ∇w is the gradient of the moisture field;
- t is time [s].

#### 2.4.2. Diffusivity Tensor and Local Rotation

The diffusivity tensor takes the form [19,34,35]:

(5) $$D\left(w\right)=\left[\begin{array}{cc} D_{xx}\left(w\right) & D_{xy}\left(w\right)\\ D_{yx}\left(w\right) & D_{yy}\left(w\right) \end{array}\right]\;,\;\;with\;\;D_{xy}=D_{yx}$$

Each component depends nonlinearly on local moisture content:

(6) $$D_{ij}\left(w\right)=D_{ij}^{0}\cdot {\left(\frac{w}{w_{sat}}\right)}^{n}$$

To model orientation-dependent behavior (e.g., fiber-reinforced or layered materials), the local diffusivity tensor is rotated:

(7) $$D_{rot}=R^{T}\cdot D_{local}\cdot R$$

where R is the rotation matrix:

(8) $$R=\left[\begin{array}{cc} \cos\theta & \sin\theta \\ -\sin\theta \; & \cos\theta \end{array}\right]$$

with θ being the material orientation angle relative to the global coordinate system.

#### 2.4.3. Numerical Discretization (Explicit Scheme)

The computational domain is discretized using a regular 2D grid with $N_{x}\times N_{y}$ nodes. The governing equation is discretized using a first-order forward Euler scheme in time and second-order central differences in space.

The updated moisture content in a generic internal node $\left(i,j\right)$ is given by [19,35]:

(9) $$w_{i,j}^{n+1}=w_{i,j}^{n}+\Delta t\cdot \left(\frac{\partial }{\partial x}\left(D_{xx}\frac{\partial w}{\partial x}\right)+\frac{\partial }{\partial y}\left(D_{yy}\frac{\partial w}{\partial y}\right)+2Dxy\cdot \frac{\partial^{2}w}{\partial x\partial y}\right)$$

This is approximated using:

(10) $$\frac{\partial^{2}w}{\partial x^{2}}\approx \frac{w_{i,j+1}-2w_{i,j}+w_{i,j-1}}{\Delta x^{2}}$$

(11) $$\frac{\partial^{2}w}{\partial y^{2}}\approx \frac{w_{i+1,j}-2w_{i,j}+w_{i-1,j}}{\Delta y^{2}}$$

(12) $$\frac{\partial^{2}w}{\partial x\partial y}\approx \frac{w_{i+1,j+1}-w_{i-1,j+1}-w_{i+1,j-1}+w_{i-1,j-1}}{4\Delta x\Delta y}$$

This scheme is conditionally stable. The time step Δt must satisfy a Couranta–Friedrichsa–Lewy (CFL)-like condition:

(13) $$\alpha_{x}=D_{xx}\frac{\Delta t}{\Delta x^{2}},\;\;\alpha_{y}=D_{yy}\frac{\Delta t}{\Delta y^{2}},\;\;\alpha_{x}+\alpha_{y}<0.5$$

#### 2.4.4. Boundary Conditions

Accurate modeling of moisture transport in porous materials requires not only a reliable constitutive formulation but also a physically meaningful definition of boundary conditions. Since building components often interact with highly variable environmental exposures, such as permanent contact with wet soil, intermittent rain, or moisture exchange with ambient air, the correct representation of these interactions at the boundaries is crucial for realistic predictions. Below is a list of possible boundaries:

Dirichlet condition (e.g., wall in contact with wet soil) [17,19,35]:

(14) $$w\left(x=0,y,t\right)=w_{A}$$

Robin-type condition (e.g., moisture exchange with air) [17,19,36]:

(15) $$-D_{ii}\cdot \frac{\partial w}{\partial i}=h\cdot \left(w-w_{\infty }\right)\Rightarrow w_{N}=w_{N-1}+\beta \cdot \frac{w_{\infty }}{1+\beta },\;\;\beta =h\cdot \Delta i{\;D}_{ii}$$

Neumann (zero flux) [17,19,35]:

(16) $${\left.\frac{\partial w}{\partial y}\right|}_{y=0}=0,\;\;{\left.\frac{\partial w}{\partial y}\right|}_{y=H}=0$$

These boundary conditions reflect realistic physical scenarios commonly encountered in engineering practice. The Dirichlet condition on the wet side of the wall models permanent contact with a moisture source, such as saturated soil or standing water. The Robin-type boundary on the opposite side captures the moisture exchange between the concrete surface and ambient air, accounting for both material properties and environmental conditions through the surface transfer coefficient h. The adiabatic (zero-flux) conditions at the top and bottom edges represent symmetry or insulation and are appropriate when there is no significant moisture exchange across these boundaries.

#### 2.4.5. Initial Conditions

The specification of appropriate initial conditions is equally important, especially in transient simulations. In practical scenarios, the initial moisture distribution often reflects a previously equilibrated state of the material, resulting from long-term exposure to indoor or outdoor environmental conditions. In the present study, the initial state is assumed to be spatially uniform, which simplifies the numerical implementation and enables clear interpretation of the moisture ingress dynamics driven solely by boundary conditions. Here, at time t=0, the moisture field is assumed to be uniform:

(17) $$w(x,y,0)=w_{0}$$

#### 2.4.6. Numerical Model Implementation

The digital twin [37,38,39,40,41] was implemented in MATLAB (9.14. (R2023a)) [42] using custom scripts based on an explicit finite-difference scheme. This environment was selected for its flexibility in implementing nonlinear and anisotropic moisture diffusion, direct integration of experimental gravimetric and hygrometric data, and control over boundary conditions. Compared to commercial software, such as WUFI 2D, the MATLAB model achieved reduced computation times and easier adaptation for parametric studies and long-term wetting–drying simulations. The custom implementation also enabled testing of alternative constitutive relationships and facilitated transparent calibration against experimental results. Furthermore, MATLAB’s built-in visualization tools allowed for rapid comparison of simulation outputs with measured values, ensuring effective model validation.

## 3. Results

The results of the experimental tests and numerical simulations concerning moisture transport in walls made of autoclaved aerated concrete, ceramic hollow blocks, perlite concrete, concrete blocks, and Sika sand–lime blocks after a 30-day simulated exposure to rainfall are presented in Table 1 and Table 2. The aim of the analysis was to determine the vertical and depth-wise distribution of moisture within the wall assembly, as well as to evaluate the consistency between the experimental results and the predictions obtained using a digital twin model. The observed differences between materials can be attributed primarily to their pore structure and capillary absorption capacity. In AAC and perlite concrete, characterized by open micro- and mesoporous systems, strong capillary suction accelerates water uptake and leads to steep moisture gradients near the surface. This is consistent with the findings of Yu et al. [4] and with recent studies on porous concretes [43], which highlight the decisive role of pore connectivity and effective capillary radius in governing absorption kinetics. By contrast, denser silicate and concrete blocks exhibit more limited capillary transport, where moisture ingress occurs mainly through surface adsorption and slower vapor diffusion [2,22]. The comparative results obtained here are therefore in line with the reported literature values for equilibrium moisture content and absorption coefficients of these materials [3,34].

### 3.1. Results of Oven-Drying (Gravimetric) Measurements

Moisture content measurements performed using the oven-drying method revealed a clear moisture gradient across the wall cross-section. The highest moisture values were recorded in the near-surface layer (approximately 5 cm), with moisture levels decreasing with material depth. The measurement was carried out at a height of 35 cm above the floor, at a temperature of 19.3 °C and relative indoor humidity of 42.1%. The measurement depths of 5 cm and 12.5 cm were selected to capture the contrast between the near-surface zone, which is most strongly influenced by capillary suction and direct contact with water, and the deeper zone, where moisture transport is governed primarily by slower diffusion processes. This approach allowed us to evaluate both the immediate and delayed response of the material to wetting.

### 3.2. Results of Non-Destructive (Electrical) Measurements

Measurements taken using three different moisture meters—dielectric, resistance-based, and microwave—are presented in Table 2. The measurements were conducted at heights of 10 cm, 40 cm, and 60 cm above the floor, and at the following depths: 1 cm for the Brennenstuhl MD device, 4 cm for the Gann meter, and 30 cm for the Trotec T610. The ambient temperature during the measurements was 19.3 °C, and the relative humidity in the room was 42.1%.

Non-destructive measurements (Table 2) revealed substantial differences between materials. The highest moisture levels were observed in the walls made of autoclaved aerated concrete, and perlite concrete, which correlates with the gravimetric results. Interestingly, elevated readings from the T610 and Gann meters were recorded for concrete blocks and Sika units. This may have resulted from surface moisture or calibration errors (e.g., the influence of material density on microwave sensor readings). The use of different measurement heights demonstrated the vertical distribution of moisture, which, in some cases (e.g., Sika), did not follow a consistent pattern. This may indicate that the material has lower capillarity and is more affected by surface adsorption or condensation.

### 3.3. Results for the Wall Made of Autoclaved Aerated Concrete Blocks

Due to the large volume of data requiring further interpretation and the need to verify the experimental results against numerical simulations, it was decided to focus subsequent analysis on the wall made of autoclaved aerated concrete blocks. This material was chosen because it is the most representative and the most extensively documented in the literature compared to the other materials. Table 3 presents the data exclusively for the autoclaved aerated concrete wall. The measured moisture percentages reported in Table 3 were determined using the gravimetric method according to Equation (3), based on the ratio of water mass to dry mass of the core samples.

**Figure 2:**
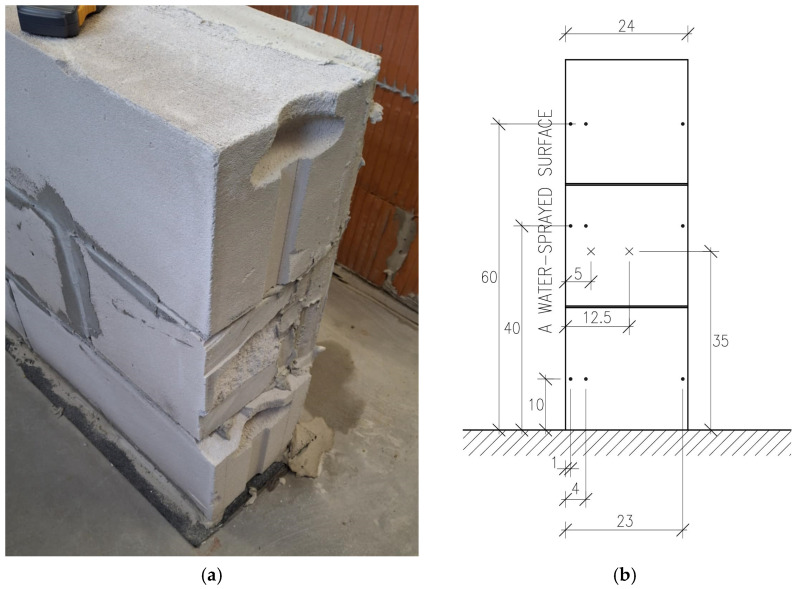
Autoclaved aerated concrete wall (**a**) and measurement point locations (**b**). Adopted symbols: • measurement points for non-destructive tests, × measurement points for destructive tests.

**Figure 3:**
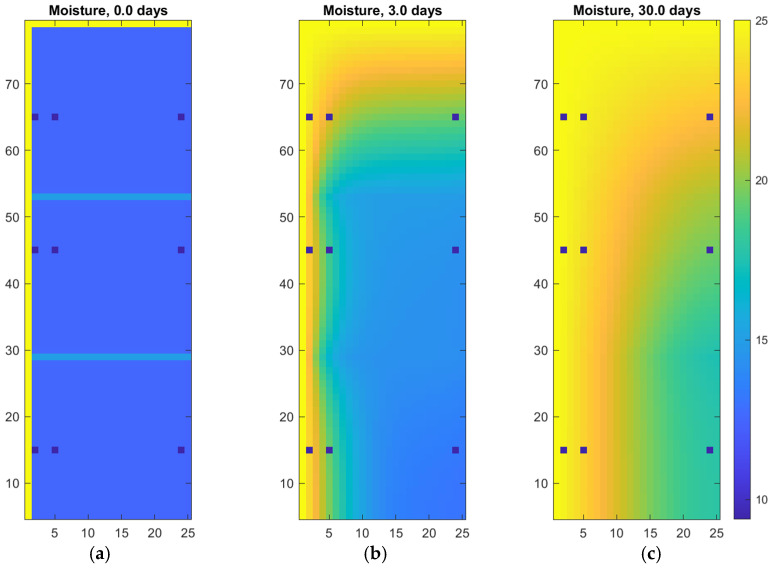
Digital twin of autoclaved aerated concrete wall: (**a**) initial and boundary conditions; (**b**) moisture penetration after 3 days; (**c**) moisture penetration after 30 days.

Figure 2 shows the autoclaved aerated concrete wall and the locations of the measurement points. Figure 3 presents the results of a 2D numerical simulation of moisture transport in a wall made of autoclaved aerated concrete blocks. The first image (0.0 days) shows the initial condition with a uniform low moisture content throughout the wall, and two visible vertical mortar joints represented as narrow layers with slightly different properties. Boundary conditions are applied on the left and top edges of the domain, simulating continuous water exposure (e.g., rainfall or rising damp), while the other edges are treated as impermeable. The yellow-highlighted regions indicate the areas in direct contact with moisture sources at the beginning of the simulation.

The subsequent images show the progression of moisture penetration after 3 days and 30 days of exposure. Water diffuses rapidly through the contact zones and gradually spreads into the deeper parts of the wall. The influence of the mortar joints is clearly visible, as they facilitate slightly faster transport due to their distinct material properties. Over time, a pronounced moisture gradient develops, with higher saturation near the wet boundaries and decreasing values toward the interior of the wall.

The results obtained from the oven-drying method (Table 1) clearly indicate a pronounced moisture gradient with respect to wall depth, particularly in the cases of autoclaved aerated concrete, perlite concrete, and ceramic hollow blocks. Moisture levels were significantly higher in the near-surface layer (5 cm), which aligns with the theory of capillary transport. It is worth noting that autoclaved aerated concrete exhibited the highest water saturation, confirming its high absorbability and highly porous structure.

Based on these findings, the wall made of autoclaved aerated concrete blocks was selected for further quantitative analysis and numerical model validation. This decision was supported by several key considerations. First, among all tested materials, autoclaved aerated concrete showed the highest level of moisture accumulation in both the surface and deeper layers, making it particularly susceptible to moisture-related effects in real-world conditions. Second, the moisture distribution across the wall section was the most distinct and interpretable, with a clear vertical and depth-wise gradient, allowing for detailed analysis of moisture transport over time. Third, this material is widely used in modern residential construction, and its hygrothermal properties are well-documented in the literature, facilitating comparison between experimental and simulation data with reference benchmarks. Finally, due to its porous structure, autoclaved aerated concrete presents a demanding test case for the digital twin, enabling evaluation of the model’s ability to reproduce complex nonlinear and directional moisture transport processes within the material.

**Figure 4:**
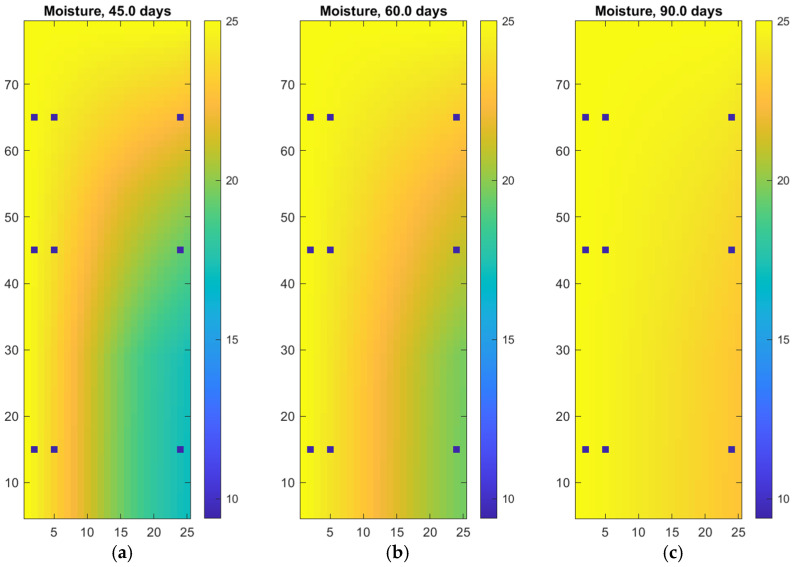
Digital twin of autoclaved aerated concrete wall: (**a**) moisture penetration after 45 days; (**b**) moisture penetration after 60 days (**c**) moisture penetration after 90 days.

The extended simulation results for 45, 60, and 90 days illustrate the long-term behavior of moisture transport in an autoclaved aerated concrete wall (see Figure 4 and Table 4), as predicted by the numerical model beyond the typical timeframe of laboratory experiments. These figures highlight the gradual saturation of the wall due to prolonged exposure to external moisture sources. Over time, the moisture front advances deeper into the structure, and the previously pronounced gradient becomes more uniform, indicating that the wall is approaching a quasi-steady moisture distribution.

This modeling approach demonstrates its practical value not only in estimating moisture absorption times but also in forecasting drying periods under various boundary conditions. By accurately capturing both the kinetics of moisture ingress and the role of different material layers (e.g., joints, coatings), the tool serves as a powerful aid for assessing long-term moisture behavior in building envelopes. Its predictive nature makes it especially useful in the design of durable wall assemblies and in evaluating the effectiveness of protective layers or rehabilitation strategies without the need for extended physical testing.

**Figure 5:**
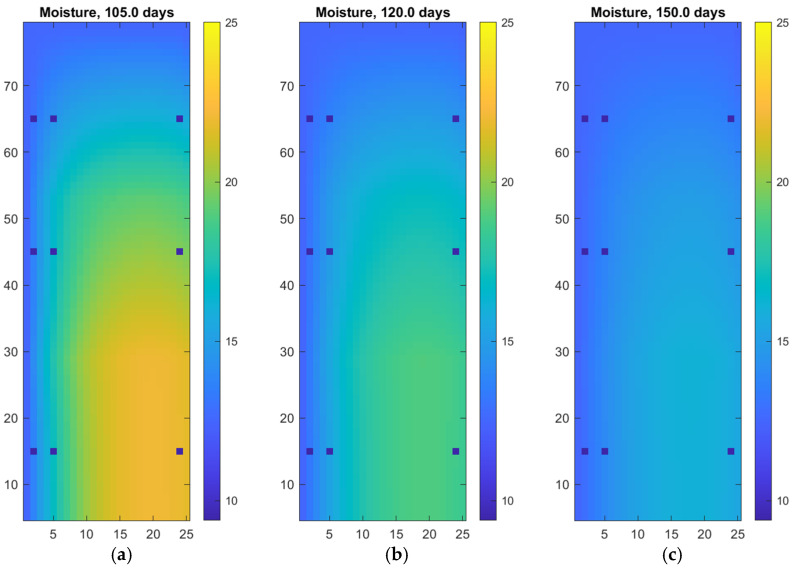
Digital twin of autoclaved aerated concrete wall: (**a**) drying process after 15 days; (**b**) drying process after 30 days (**c**) drying process after 60 days.

Figure 5 illustrates the drying phase of the wall after prior long-term exposure to moisture. Between 105 and 150 days, we observe a gradual reduction of moisture content throughout the structure. Initially, at 105 days, the highest moisture levels are still visible in the lower-left region, indicating the previous moisture source. As time progresses, moisture redistributes and decreases, driven by internal diffusion and surface exchange with drier ambient air.

By 60 days (after 90 days of intensive rain), the entire wall shows a relatively uniform and low moisture profile (see Table 5), indicating that the drying process is well advanced. The initially steep gradients have leveled out, and the system is approaching hygrothermal equilibrium. This confirms that the numerical model can effectively simulate not only moisture ingress but also drying dynamics, providing valuable insight into the long-term moisture behavior of wall systems under changing boundary conditions.

## 4. Discussion

The results of the conducted study clearly confirm the high effectiveness of integrating experimental methods with digital modeling (digital twin) in predicting moisture transport in building materials. Particularly important was the agreement between the numerical simulation results and the data obtained from both oven-drying and non-destructive methods, which demonstrates the validity of the adopted model in replicating real operating conditions. It should be emphasized that the study was performed under stable laboratory conditions, with constant temperature and relative humidity. While this setting ensured reproducibility and precise calibration of the digital twin, it does not fully replicate the variability of outdoor climates, where factors such as wind-driven rain, solar radiation, freeze–thaw cycles, and diurnal fluctuations significantly affect moisture transport. Although absolute moisture contents may differ under such real-world conditions, the main trends observed in AAC—namely high capillarity, strong surface saturation, and depth-dependent gradients—remain valid. Importantly, the digital twin framework is designed to accommodate variable boundary conditions, and future extensions will integrate real climate data to broaden its practical applicability.

Consistent with previous research [1,3,17], the wall made of autoclaved aerated concrete showed the highest susceptibility to moisture accumulation, which is attributed to its high porosity, low bulk density, and strong capillarity. Studies presented in [1,3] also indicate that materials with micro- and mesoporous structures exhibit rapid water saturation under prolonged moisture exposure, with a clear depth-dependent gradient. Similarly, the authors of [4] observed that under simulated rainfall conditions, porous materials exhibit uneven moisture absorption, which may lead to localized material overload and degradation risk.

The digital twin model developed in this study incorporates the nonlinearity of the relationship between moisture content and diffusion coefficient, which is supported by numerous studies [19,21,34], demonstrating that classical linear models (e.g., Fick’s second law) are insufficient for describing the complex moisture transport processes in cement-based materials. Studies [17,34] have shown that introducing material anisotropy (e.g., due to reinforcement, layer orientation, or microcracking) significantly improves the accuracy of moisture migration predictions under complex operating conditions.

Despite their practical advantages, the non-destructive methods showed some deviations from the reference method (oven-drying), which may be attributed to calibration limitations and the influence of dielectric properties and material density on the readings [2,30]. This issue has also been raised by the authors of [22], who emphasized the need for material-specific calibration of microwave and resistance meters. Therefore, results obtained using non-invasive methods should always be complemented by direct measurements, particularly when calibrating numerical models.

Compared to existing tools, the proposed digital twin significantly extends analytical capabilities. In contrast to conventional software, such as WUFI 2D [20], the model developed in this study offers greater flexibility and shorter computation times while maintaining high predictive accuracy, making it a useful tool for material analysis, envelope durability prediction, and building design under various climatic conditions. A similar approach was adopted in [25,27], where the importance of the digital twin as a design and diagnostic tool in civil engineering was emphasized.

A key limitation of the present study is that the experiments were conducted under laboratory conditions with relatively stable environmental parameters. As noted in [5], real-world exterior conditions are characterized by high variability and nonlinearity, with factors such as solar radiation, wind, and freeze–thaw cycles having a significant impact on moisture transport and material degradation. Consequently, future studies are planned to include variable climatic conditions, thermal effects, and long-term operational scenarios, in line with recommendations in standards such as EN 15026 and International Organization for Standardization (ISO) 13788 [11,36].

The digital twin [37,38,39,40,41] developed in this study was calibrated exclusively for autoclaved aerated concrete (AAC), reflecting its high porosity and vulnerability to moisture-induced degradation. While other materials were tested experimentally for comparison, the numerical framework was tailored to AAC, and future work will extend this approach to additional wall systems. To the best of our knowledge, this is the first digital twin of AAC that integrates calibration with both gravimetric and hygrometric data, while also incorporating nonlinear and anisotropic formulations of moisture diffusion. These features enhance the model’s ability to replicate real-world hygrothermal behavior beyond the scope of traditional steady-state or linear approaches. From a practical perspective, such a tool can guide material selection in humid climates by identifying configurations most resistant to long-term saturation. Moreover, the framework has the potential to be integrated into BIM workflows and real-time monitoring systems, supporting predictive maintenance, durability assessment, and the design of resilient building envelopes.

The reliability of the digital twin was strengthened by calibration against multiple AAC specimens (*n* = 6), which minimized the influence of single-sample variability. As a result, the model reproduces moisture transport trends with greater confidence, and the agreement between simulations and measurements reflects not only individual behavior but also consistent material response under repeated testing. In conclusion, the present research confirms the high effectiveness of using a digital twin model to predict moisture transport in building materials. The integrated approach combining experimental and numerical data provides a modern tool for diagnostics, design, and optimization of building structures with regard to durability and moisture resistance.

## 5. Conclusions

The conducted study demonstrated that the wall made of autoclaved aerated concrete reached a state of sustained moisture saturation after 30 days of exposure to simulated rainfall, classifying it as a “wet” wall. The moisture content determined by the oven-drying method exceeded 19% by mass in the near-surface layer and 18% in the deeper layers, significantly surpassing the threshold values for safe operating conditions commonly cited in the literature and standards. For porous materials, such as autoclaved aerated concrete, exceeding 15% moisture content is already indicative of a near-saturation state, which promotes material degradation, deterioration of thermal performance, and the growth of microorganisms. The walls made of perlite concrete and ceramic hollow blocks also showed clear signs of water saturation, reaching values typical for wet materials. In contrast, walls made of concrete blocks and Sika, despite showing locally elevated surface moisture levels, did not meet the criteria for sustained saturation and can be classified as damp, but not wet.

All tested materials exhibited a distinct moisture gradient with depth, consistent with the mechanism of capillary moisture transport under unidirectional wetting conditions. Non-destructive measurements confirmed the general trends in moisture distribution, although some discrepancies in values were observed due to material properties and calibration limitations of the instruments. The use of measurements at multiple heights proved especially valuable, as it enabled assessment of vertical moisture migration and identification of zones with increased risk of moisture accumulation.

The experimental data obtained provide a critical basis for assessing the technical condition of building envelopes and validating the digital twin model. Integrating the measurement results with the numerical model allowed for realistic representation of moisture transport processes, accounting for the nonlinearity and anisotropy of material properties. The developed model can be effectively applied to evaluate envelope durability, predict the risk of material degradation, and support design decisions regarding the selection of moisture-resistant materials under varying operational conditions.

## Figures and Tables

**Table 1:** Results of moisture content measurement using the oven-drying (gravimetric) method (MC [%]).

Measurement Depth	Autoclaved Aerated Concrete Wall	Ceramic Hollow Block Wall	Perlite Concrete Wall	Concrete Block Wall	Sika Block Wall
5 cm	19.73	2.72	11.70	1.19	1.50
12.5 cm	18.65	2.30	14.04	2.01	3.98

**Table 2:** Results of moisture content measurement using non-destructive methods.

Height of Measurement Above Floor Level	Instrument Used	Autoclaved Aerated Concrete Wall	Ceramic Hollow Block Wall	Perlite Concrete Wall	Concrete Block Wall	Sika Block Wall
10 cm	T610 [jtk]	15.7	12.4	19.9	39	35.8
	MD [%]	23.6	15.7	22.9	8.3	7.8
	Gann [jtk]	130	110	129	98	130
40 cm	T610 [jtk]	18	37.6	27.6	34.6	25.3
	MD [%]	24	18.5	24.6	15	17
	Gann [jtk]	134	112	130	102	132
60 cm	T610 [jtk]	23.9	23.9	25.6	33	38
	MD [%]	24	19.5	23.6	19.8	20.4
	Gann [jtk]	136	118	134	119	133

**Table 3:** Measured and computed moisture in the autoclaved aerated concrete wall.

Vertical Location of Measurement	Measurement Depth	Measured Moisture [%]	Computed Moisture [%]
10 cm	1 cm	23.6	24.3
	4 cm	21.3	22.3
	23 cm	15.7	15.2
40 cm	1 cm	24.0	24.5
	4 cm	20.1	23.0
	23 cm	18.0	17.8
60 cm	1 cm	24.0	24.8
	4 cm	24.0	24.1
	23 cm	23.9	21.7

**Table 4:** Simulated moisture for the autoclaved aerated concrete wall.

Vertical Location of Measurement	Measurement Depth	Moisture After 45 Days [%]	Moisture After 60 Days [%]	Moisture After 90 Days [%]
10 cm	1 cm	24.6	24.8	24.9
	4 cm	23.4	24.1	24.7
	23 cm	17.3	19.7	22.9
40 cm	1 cm	24.7	24.8	24.9
	4 cm	23.9	24.3	24.8
	23 cm	19.4	21.1	23.4
60 cm	1 cm	24.9	24.9	24.9
	4 cm	24.5	24.7	24.9
	23 cm	22.5	24.2	24.2

**Table 5:** Drying simulated after 90 days of intensive rain for the autoclaved aerated concrete wall.

Vertical Location of Measurement	Measurement Depth	Moisture After 105 Days [%]	Moisture After 120 Days [%]	Moisture After 150 Days [%]
10 cm	1 cm	13.8	13.3	12.9
	4 cm	17.0	15.3	14.0
	23 cm	21.8	18.6	15.6
40 cm	1 cm	13.7	13.2	12.8
	4 cm	16.6	14.9	13.7
	23 cm	19.9	17.3	14.9
60 cm	1 cm	13.1	12.8	12.6
	4 cm	14.5	13.6	13.1
	23 cm	15.9	14.6	15.5

## Data Availability

The original contributions presented in this study are included in the article. Further inquiries can be directed to the corresponding author.
